# Opportunities for the CTEI: disentangling frequency and quality in evaluating teaching behaviours

**DOI:** 10.1007/s40037-012-0023-2

**Published:** 2012-09-18

**Authors:** Johanna Schönrock-Adema, Peter M. Boendermaker, Pine Remmelts

**Affiliations:** 1 Center for Research and Innovation in Medical Education, University of Groningen and University Medical Center Groningen, Antonius Deusinglaan 1, 9713 AV Groningen, the Netherlands; 2 Wenckebach Institute, University of Groningen and University Medical Center Groningen, Groningen, the Netherlands

**Keywords:** CTEI, Evaluation, Teaching behaviours, Teaching quality

## Abstract

Students’ perceptions of teaching quality are vital for quality assurance purposes. An increasingly used, department-independent instrument is the (Cleveland) clinical teaching effectiveness instrument (CTEI). Although the CTEI was developed carefully and its validity and reliability confirmed, we noted an opportunity for improvement given an intermingling in its rating scales: the labels of the answering scales refer to both frequency and quality of teaching behaviours. Our aim was to investigate whether frequency and quality scores on the CTEI items differed. A sample of 112 residents anonymously completed the CTEI with separate 5-point rating scales for frequency and quality. Differences between frequency and quality scores were analyzed using paired *t* tests. Quality was, on average, rated higher than frequency, with significant differences for ten out of 15 items. The mean scores differed significantly in favour of quality. As the effect size was large, the difference in mean scores was substantial. Since quality was generally rated higher than frequency, the authors recommend distinguishing frequency from quality. This distinction helps to obtain unambiguous outcomes, which may be conducive to providing concrete and accurate feedback, improving faculty development and making fair decisions concerning promotion, tenure or salary.

## Introduction

Students’ perceptions of teaching quality are vital for quality assurance purposes [1–4]. Optimizing teaching quality may not only result in better student learning outcomes, but also in higher quality educational programmes for the institution and improved patient care [5]. Within medical education, clinical teaching effectiveness has therefore received a lot of attention. Efforts to measure teaching effectiveness adequately include attempts to identify the characteristics of effective clinical teachers [3, 6, 7]. Examples of characteristics regarded important for effective teaching are, for example, establishing a positive learning climate, modelling competencies, and providing feedback on a regular basis.

One widely used, generic (i.e. department-independent) questionnaire for measuring teaching quality is the (Cleveland) clinical teaching effectiveness instrument (CTEI) [3]. The items of the CTEI were developed following a conscientious qualitative procedure. A first investigation using the CTEI indicated that the CTEI is a reliable, valid and usable instrument with good content validity [3]. Several studies confirmed the reliability and the validity of the CTEI [3, 4, 8–13].

Despite the careful development process applied, the CTEI might benefit from an adjustment, given an intermingling that we noticed in its rating scales. We observed that the labels of the answering scales concern both the frequency and the quality of teaching behaviours, for example, ‘never/poor’ and ‘always/superb’. Consequently, the items and their responses are multi-interpretable as they can refer to both qualitative and quantitative aspects of the teaching behaviours in question. Findings by the developers of the CTEI––Copeland and Hewson––corroborate this view: they found that most variance in their CTEI data was attributable to the interaction between raters and items, implying that raters interpreted items differently [3]. This finding may, at least partly, be attributable to the ambiguity in the rating scales. It can be reasoned that the ambiguity in the rating scales may lead to inconsistent ratings. Imagine, for example, a teacher who displays good supervising skills, but lacks the time to supervise frequently. If this teacher is judged on the quality of teaching, he will receive high ratings and positive feedback, whereas he will receive relatively low ratings and more criticism if he is judged on frequency of teaching. Hence, it can be concluded that the intermingling in rating scales may decrease the usefulness of the ratings.

Addressing quality and quantity of educational activities separately may increase transparency for respondents and increase the interpretability and, hence, the usefulness of the ratings. In addition, it may help to increase the specificity of feedback, one of the key elements of effective feedback [14–17]. Discriminating between frequency and quality particularly adds to the quality of the CTEI if respondents assign different scores for both of these aspects. Therefore, the aim of this study was to investigate whether frequency and quality scores differed. Since we do not find it credible that these scores will be similar, our hypothesis was that frequency scores differ from scores pertaining to the perceived quality of these behaviours.

## Method

### Respondents and procedure

A sample of 112 residents anonymously completed the CTEI with adjusted rating scales. The respondents were instructed to arbitrarily choose a teacher who supervised them during the past 3 months and to assess his or her teaching performance. As they did not have to reveal which supervisor they chose for assessment, complete anonymity of both raters and ratees was guaranteed. In addition to the fact that neither respondents nor supervisors can be identified from the data presented, we would like to emphasize that no plausible harm to participating individuals arises from this study. To control for rating sequence, we randomly distributed four versions of the CTEI––differing in sequence of items and rating scales––across the respondents (see “Instrument”).

### Instrument

The (Cleveland) CTEI is an evaluation tool for rating teaching effectiveness in a wide variety of clinical teaching settings that contains 15 items on a 5-point scale (1 = never/poor, 5 = always/superb). In this study, we used the Dutch version of the CTEI which was approved by the original developers [10]. We adjusted its rating scales by discriminating between frequency scores and quality scores: in our study, all 15 items had to be rated on both a frequency and a quality scale. Therefore, two 5-point rating scales were inserted behind each item. To approximate the requirement of equal intervals between scale points and have the scales evenly distributed, we used discrete visual analogue scales, which means that we only labelled the poles of the rating scales [18]. The poles of the frequency and quality scales were labelled 1 = ‘*never*’ and 5 = ‘*always*’, and 1 = ‘*very poor*’ and 5 = ‘*very good*’ respectively. As one of the 15 items contained a reference to frequency (‘*regularly* gives feedback, both positive and negative’), we removed the word *regularly*. To control for possible effects of item and scale sequence, we constructed four versions. The order of the 15 CTEI items in versions C and D was reversed compared with the order in versions A and B. Additionally, in versions A and C the items were first followed by the frequency scale and then by the quality scale, whereas in versions B and D this order was reversed.

### Data analysis

The differences between frequency and quality of teacher performance were statistically analyzed using paired *t* tests. We calculated the effect size (*r*) to find out whether differences were substantial, with the thresholds for small, medium and large effects being *r* = 0.10, *r* = 0.30 and *r* = 0.50, respectively [19].

## Results

### Descriptives

The internal consistencies of the frequency scale and the quality scale were high with Cronbach’s alphas of 0.80 and 0.84, respectively. The correlations between frequency and quality scores on the items ranged from 0.37 to 0.68 (*p* < 0.001) and the correlation between the mean frequency and quality scores of the items was 0.69 (*p* < 0.001). The percentages of respondents who assigned different scores for frequency and quality of teaching behaviours ranged from 27.8 % for item 1 *Establishes a good learning environment* to 49 % for item 11 *Coaches me on my clinical/technical skills* (Table 1). For 13 of the 15 items, quality was rated higher than frequency.

**Table 1:** Absolute differences between frequency and quality CTEI scores

		Absolute differences between frequency and quality scores (%)					Differences between frequency (f) and quality (q) scores (%)		
		0	1	2	3	4	f < q	f = q	f > q
1.	Establishes a good learning environment (approachable, non-threatening, enthusiastic, etc.)	72.2	23.1	2.8	1.9	–	13.0	72.2	14.8
2.	Stimulates me to learn independently	59.4	33.0	3.8	3.8	–	25.5	59.4	15.1
3.	Allows me autonomy appropriate to my level/experience/competence	60.6	32.7	3.8	2.9	–	14.4	60.6	25.0
4.	Organizes time to allow for both teaching and care giving	59.0	30.5	6.7	3.8	–	23.8	59.0	17.1
5.	Offers feedback (both positive and negative)	60.0	21.8	15.5	2.7	–	29.1	60.0	10.9
6.	Clearly specifies what I am expected to know and do during the training period	59.4	34.0	6.6	–	–	28.3	59.4	12.3
7.	Adjusts teaching to my needs (experience, competence, interest, etc.)	61.0	32.4	5.7	1.0	–	25.7	61.0	13.3
8.	Asks questions that promote learning (clarifications, probes, reflective questions, etc.)	59.8	27.1	10.3	2.8	–	30.8	59.8	9.3
9.	Gives clear explanations/reasons for opinions, advice actions, etc.	59.3	30.6	7.4	2.8	–	25.9	59.3	14.8
10.	Adjusts teaching to diverse settings (bedside, view box, OR, consultation room, etc.)	63.0	29.0	8.0	–	–	29.0	63.0	8.0
11.	Coaches me on my clinical/technical skills (interview, diagnostic, examination, procedural, laboratory, etc.)	51.0	35.3	9.8	3.9	–	41.2	51.0	7.8
12.	Incorporates research data and/or practice guidelines into teaching	61.9	34.3	1.9	1.0	1.0	29.5	61.9	8.6
13.	Teaches diagnostic skills (clinical reasoning, selection/interpretation of tests, etc.)	59.8	28.0	12.1	–	–	30.8	59.8	9.3
14.	Teaches effective patient and/or family communication skills	56.3	30.2	10.4	3.1	–	35.4	56.3	8.3
15.	Teaches me principles of cost-appropriate care (resource utilization, etc.)	65.7	26.3	8.1	–	–	28.3	65.7	6.1
Average		60.6	29.9	7.5	2.0	0.1	27.4	60.6	12.0

### *T* tests

The differences in frequency and quality scores were significant for ten of the 15 items, with all differences in favour of quality (Table 2). Four of these differences were of medium effect size (> 0.30). The other six differences in favour of quality were small (effect sizes > 0.10). The differences between the mean scores on frequency and quality were significant (*t*(67) = −5.17, *p* < 0.001), and relevant with an effect size of *r* = 0.53, which is large and therefore represents a substantive finding [19].

**Table 2:** Differences in mean CTEI scores between frequency and quality

		Frequency		Quality		*t*	df	*p*	ES
		M	SD	M	SD				
1.	Establishes a good learning environment (approachable, non-threatening, enthusiastic, etc.)	4.03	0.90	4.02	0.89	0.134	107	n.s.	–
2.	Stimulates me to learn independently	3.74	1.00	3.84	0.91	−1.182	105	n.s.	–
3.	Allows me autonomy appropriate to my level/experience/competence	4.24	0.73	4.15	0.80	1.026	103	n.s.	–
4.	Organizes time to allow for both teaching and care giving	3.46	0.89	3.53	0.98	−0.815	104	n.s.	–
5.	Offers feedback (both positive and negative)	3.35	0.97	3.65	0.93	−3.145	109	<0.01	0.29
6.	Clearly specifies what I am expected to know and do during the training period	3.20	0.88	3.37	0.88	−2.295	105	<0.05	0.22
7.	Adjusts teaching to my needs (experience, competence, interest, etc.)	3.56	0.87	3.72	0.81	−2.111	104	<0.05	0.20
8.	Asks questions that promote learning (clarifications, probes, reflective questions, etc.)	3.54	1.00	3.82	0.82	−3.120	106	<0.01	0.29
9.	Gives clear explanations/reasons for opinions, advice actions, etc.	3.86	0.81	3.95	0.86	−1.043	107	n.s.	–
10.	Adjusts teaching to diverse settings (bedside, view box, OR, consultation room, etc.)	3.54	0.87	3.77	0.72	−3.066	99	<0.01	0.29
11.	Coaches me on my clinical/technical skills (interview, diagnostic, examination, procedural, laboratory, etc.)	3.41	0.90	3.86	0.82	−4.792	101	<0.001	0.43
12.	Incorporates research data and/or practice guidelines into teaching	3.61	0.83	3.77	0.79	−2.079	104	<0.05	0.20
13.	Teaches diagnostic skills (clinical reasoning, selection/interpretation of tests, etc.)	3.52	0.82	3.80	0.73	−3.481	106	<0.001	0.32
14.	Teaches effective patient and/or family communication skills	3.00	0.93	3.38	0.92	−3.943	95	<0.001	0.38
15.	Teaches me principles of cost-appropriate care (resource utilization, etc.)	3.00	0.86	3.26	0.78	−3.616	98	<0.001	0.34
Mean scores		3.57	0.45	3.79	0.43	−5.167	67	<0.001	0.53

## Discussion

Our study confirmed that ratings of the frequency of teaching behaviours differ from those of their quality. In general, quality scores were higher than frequency scores. The mean differences were even large [19]. The current findings suggest that separating frequency from quality may add to the quality of the CTEI. Besides, measuring both quantity and quality of behaviours complies with the recommendations of the Association of American Medical Colleges [20, 21]. Disentangling frequency from quality yields transparent and unambiguously interpretable scores, which implies an improvement of the validity of the instrument (‘does the instrument measure what it should measure?’) and, hence, of the usefulness of the data. In addition, it may help to increase the specificity of feedback, which is important to the effectiveness of the feedback [14–17]. In turn, this increased specificity may help to gear further training towards the individual needs of teachers and thus improve faculty development [5]. Increased transparency due to separating frequency from quality may also improve the comparability of teacher performance, which is important if the information obtained is to be used for (underpinning or justifying) higher-stakes summative decisions concerning, for example, promotion, tenure or salary [22].

A limitation of this study is that we did not compare the responses on the separate rating scales with those on the original CTEI. However, such an approach may yield some problems. On the one hand, asking respondents to complete the original and the adjusted version of the CTEI bears the risk that completing one version influences scoring on the other version. On the other hand, comparing the scores of both versions by having two independent groups of respondents completing one version of the CTEI carries the risk of a confounding factor as the comparison may relate to the groups instead of the rating scale. Therefore, the present method seemed the best possible approach.

The finding that, in general, lower scores were assigned for the frequency of teaching behaviours may create the impression that teachers score better on quality than on frequency. However, our findings do not reveal which scores on frequency and on quality represent satisfactory or dissatisfactory teaching performance. Although the scales are the same (5-points), the cut-off points between sufficient and insufficient teaching performance may be different for frequency and quality. A lower score on frequency, for example, may be as satisfying as a higher score on quality. Future research is needed to set standards for sufficient teaching performance with respect to frequency and quality.

The differences found confirm that separate scales may lead to more specific and accurate feedback. In view of our outcomes, it can be hypothesized that separating frequency from quality reduces variance in the data due to interaction between raters and items. Future research should investigate whether this assumption is true and whether distinguishing between frequency and quality adds to the validity of the CTEI. We conclude that distinguishing frequency from quality of teaching behaviours seems to be an appropriate improvement of the CTEI, which may enhance its validity and practical usefulness. Therefore, we recommend the use of separate scales for frequency and quality when evaluating teachers’ behaviours.

### Essentials

- The quality of teaching performance is essential to medical education quality and, ultimately, to patient care.
- In order to be effective, feedback on teaching behaviour should be specific.
- Avoid intermingling of rating scales.
- When applying the CTEI, use separate rating scales for frequency and quality.
